# Immune checkpoint gene-based tumor-immune phenotypes stratify prognosis and immunotherapy-associated remodeling in cutaneous melanoma: a multi-cohort transcriptomic and single-cell re-analysis

**DOI:** 10.3389/fgene.2026.1978135

**Published:** 2026-10-23

**Authors:** Qing Mao, Shihu Yang, Mengtong Gong

**Affiliations:** 1 Department of Dermatology, The People’s Hospital of YueChi County, Guang’an, Sichuan, China; 2 Department of Dermatology, People’s Hospital Affiliated to Shandong First Medical University, Jinan, Shandong, China; 3 Department of Plastic Sur gery, Xuzhou Central Hospital, Southeast University, Xuzhou, Jiangsu, China

**Keywords:** cutaneous melanoma, immune checkpoint genes, immune signatures, immunotherapy-associated remodeling, overall survival, single-cell RNA sequencing, transcriptomics, tumor-immune interface

## Abstract

**Background:**

Immune checkpoint inhibitor responses in cutaneous melanoma are heterogeneous. We investigated immune checkpoint gene (ICG)-based tumor phenotypes, their immune and survival associations, treatment-associated transcriptional changes, and interferon-γ (IFN-γ)-responsive gene expression in melanoma cell lines.

**Methods:**

We clustered 47 immune checkpoint genes in 472 TCGA-SKCM tumors and assessed survival using one tumor per patient. Cox models adjusted for AJCC stage, age and sex, with a stage-stratified sensitivity model. GSE91061 comprised 109 RNA-seq samples from 65 patients, including 43 paired pretreatment/on-treatment cases. We reanalyzed all 16,291 GSE120575 immune cells using the deposited log2(TPM+1) values, aggregated expression and composition within 48 biopsies from 32 patients, and accounted for repeated patients, time point and therapy. Canonical marker plots and full gene-set definitions were supplied. Cell-line RT-qPCR assessed CD274, CXCL10 and HLA-A expression after recombinant human IFN-γ treatment at 10 or 100 ng/mL for 24 or 48 h (PeproTech), with four independent biological replicates measured in technical triplicate.

**Results:**

ICG-high, ICG-mid and ICG-low phenotypes included 136, 203 and 133 tumors. The ICG score correlated with the CD8 T-cell signature (Spearman ρ = 0.828, P = 5.06 × 10^-120^). In 454 evaluable unique patients, median overall survival was 4930, 2192, 1487 days (log-rank P = 1.50e-05). Among 404 complete cases with 190 deaths, ICG-high versus ICG-low was associated with survival after adjustment (HR 0.34, 95% CI 0.22–0.51; P = 2.68e-07), with a concordant stage-stratified estimate (HR 0.35, 95% CI 0.23–0.53; P = 6.40e-07). Paired on-treatment ICG scores increased (median change 0.115; P = 0.012). At the biopsy level, immune-cell IFN-α and IFN-γ scores were higher in nonresponders after adjustment for time point and therapy (P = 1.29e-04 and P = 0.004, respectively).

**Conclusion:**

ICG phenotypes were associated with immune-expression profiles and overall survival after adjustment for AJCC stage, age and sex. Paired bulk transcriptomes showed increased IFN-related expression during treatment, whereas the single-cell cohort showed higher immune-cell IFN scores in nonresponders. Together, these findings describe the relationship of checkpoint and IFN programs to immune context, treatment time and clinical outcome.

## 1 Introduction

Cutaneous melanoma is an aggressive skin malignancy with a substantial disease burden (Siegel et al., 2024; Arnold et al., 2022). Immune checkpoint inhibitors (ICIs) targeting the PD-1/PD-L1 and CTLA-4 pathways can produce durable responses, yet benefit varies among patients (Robert et al., 2019; Larkin et al., 2019; Wolchok et al., 2022; Kluger et al., 2020). Immune phenotypes that integrate multiple molecular features may help describe this heterogeneity. This study examines their associations with prognosis and treatment-related immune profiles. Although ICI therapy has changed the management of advanced melanoma, differences in the tumor microenvironment may contribute to variable clinical outcomes. A single checkpoint molecule may not fully capture the diversity of immune states within tumors. Integrating multiple checkpoint-related transcripts may therefore provide a broader description of the immune context and support comparisons across patient groups.

The tumor-immune interface comprises malignant cells, infiltrating immune populations, and regulatory signals that change during disease progression and treatment. Immune checkpoint molecules such as PD-1, PD-L1, CTLA-4, LAG-3, TIM-3, TIGIT, and VISTA participate in the regulation of antitumor immunity (Anderson et al., 2016; Wei et al., 2024). Their coordinated expression can accompany both immune-cell activity and inhibitory signaling. Consequently, checkpoint expression should be interpreted in the context of broader immune programs rather than treated as a direct measure of functional resistance (Boutros et al., 2024; Hossain and Eccles, 2023). In particular, elevated inhibitory-receptor expression may occur in tumors with substantial immune infiltration, while low checkpoint expression may reflect limited immune-cell recruitment rather than an absence of immune regulation. Expression-based immune signatures can help distinguish these settings, although they do not directly measure immune-cell abundance or function. Examining checkpoint genes together with cytotoxic, interferon-related, and other immune programs may improve biological interpretation.

Genomic and transcriptomic studies have established substantial heterogeneity in cutaneous melanoma, including the BRAF, RAS, NF1, and Triple-WT genomic subtypes described by TCGA (Cancer Genome Atlas Network, 2015; Tsoi et al., 2018; Thorsson et al., 2018; Bagaev et al., 2021). Mutation annotations, immune-related expression signatures and longitudinal treatment samples characterize complementary aspects of this heterogeneity. These molecular classifications do not necessarily correspond to the same immune states, making it useful to evaluate genomic and immune-transcriptional features as complementary dimensions. Moreover, cross-sectional tumor profiles provide limited information about how immune programs change after treatment begins. Paired biopsies and single-cell datasets can add temporal and cellular context, while requiring careful distinction between tumor-wide signals and immune-cell-specific expression.

Here, we used public TCGA-SKCM data to identify immune checkpoint gene (ICG)-based tumor-immune phenotypes and examine their immune signatures, available somatic-mutation annotations, and overall survival. We then assessed paired treatment-associated transcriptomic changes in GSE91061 and analyzed immune-cell features by response at the biopsy level in GSE120575. We aimed to describe whether coordinated checkpoint-gene expression defines reproducible immune phenotypes associated with clinical outcome and to place these phenotypes in the context of treatment-associated changes. By combining bulk tumor data, longitudinal samples, immune-cell transcriptomes, and a targeted melanoma-cell IFN-γ experiment, we examined related but distinct aspects of checkpoint and interferon biology. Because the cohorts and experimental systems address different questions, the findings are interpreted as context-specific associations rather than direct evidence of treatment prediction or causality.

## 2 Methods

### 2.1 Cohorts and data

TCGA-SKCM contributed 472 tumor expression profiles from 469 patients; 66 tumors had the supplied paper-linked mutation annotations. GSE91061 is the nivolumab treatment cohort reported by Riaz et al. (Riaz et al., 2017), including patients with differing prior ipilimumab exposure; its supplied RNA-seq matrix contains 109 samples from 65 patients, with 43 paired pretreatment and on-treatment cases. GSE120575, reported by Sade-Feldman et al. (Sade-Feldman et al., 2018), contains 16,291 CD45-positive immune cells from 48 biopsies representing 32 patients receiving anti-PD-1, anti-CTLA4 or combination treatment. In GSE120575, the GEO label Pre means baseline and Post means on-treatment. Response and therapy labels were taken directly from the deposited metadata.

#### 2.1.1 Immune checkpoint gene-based phenotyping

A predefined, biologically curated panel of 47 immune checkpoint-related genes was standardized separately for each gene across tumors. Unsupervised consensus clustering was performed over k = 2–6 (100 resamplings, 80% item resampling, KMeans base clustering); based on the observed cumulative distribution function (CDF) and relative area change, k = 3 was retained for this analysis. Final clusters were assigned by average-linkage hierarchical clustering on the 1-consensus distance matrix and ordered by mean ICG z-score into ICG-high, ICG-mid, and ICG-low (Python 3.13.0; NumPy 2.1.3; pandas 2.2.3; SciPy 1.17.1; scikit-learn 1.8.0). The panel was selected to represent immune regulatory pathways; the complete gene list is provided in Supplementary Table S1.

#### 2.1.2 Immune and resistance signatures

TCGA expression was analyzed as log2(supplied gene-expression value + 1), and GSE91061 FPKM values were mapped from Entrez identifiers to gene symbols, collapsed by maximum FPKM where multiple identifiers mapped to one symbol, and transformed as log2(FPKM+1). In both bulk cohorts, the ICG score was the mean of per-gene z scores across the project-defined 47-gene checkpoint panel, standardized separately within each cohort. The 14 immune/resistance signatures and eight candidate immune-cell marker sets were custom panels defined in the project configuration; their complete membership and use in each analysis are provided in Supplementary Table S1. Bulk ssGSEA used GSEApy with min_size = 3 for immune/resistance signatures and min_size = 2 for cell-marker sets, no permutations and seed = 2026. The six eligible marker sets were CD8 T, NK, B Cell, Macrophage, Dendritic and Mast; the single-gene CD4 T and Treg panels did not meet the minimum size. These ssGSEA values quantify marker expression. In GSE120575, each custom signature was the arithmetic mean of its available genes on the deposited log2(TPM+1) scale. Single-cell checkpoint analyses used the expression of each available checkpoint gene individually. VSIR was absent from the deposited symbols, giving 46 checkpoint genes and 14 signatures, or 60 response comparisons. Official MSigDB Hallmark IFN-α (97 genes) and IFN-γ (200 genes) response sets were downloaded on 18 September 2026 (Liberzon et al., 2015) and scored separately for sensitivity analyses. Supplementary Table S1 includes the custom panels, annotation markers, archived Hallmark records and gene coverage.

### 2.2 Survival and clinical analysis

TCGA overall survival used recorded death or last-follow-up time. We selected the lexicographically first tumor barcode for each patient before outcome filtering, prioritizing primary-tumor barcodes over metastatic barcodes where both existed. The resulting table contained one tumor per patient; 454 of 469 unique patients had positive follow-up and valid event status. Kaplan–Meier estimates and log-rank comparisons used this patient-level table. The multivariable Cox model included ICG phenotype (ICG-low reference), AJCC stages I–IV (I reference), age per 10 years and sex (male reference), selected for clinical relevance. Complete-case analysis included 404 patients and 190 deaths; 50 survival-evaluable patients lacked one or more required covariates. Unadjusted models on the same complete-case population were supplied for comparison. Rank-based scaled-Schoenfeld-residual tests assessed proportional hazards. Because a stage indicator showed a nominal violation, a sensitivity model stratified the baseline hazard by AJCC stage while retaining phenotype, age and sex. A separate stage-stratified model used continuous ICG score per standard deviation. Hazard ratios and 95% confidence intervals are reported in Supplementary Table S2. Clinical-score comparisons used all tumor profiles; survival models used unique patients.

### 2.3 On-treatment remodeling (GSE91061)

For each patient with paired pre-/on-treatment samples, immune signatures and ICG score were aggregated per visit; paired changes were tested with the Wilcoxon signed-rank test (n = 43 paired patients; SciPy 1.17.1).

### 2.4 Single-cell analysis and statistics

The source GSE120575 study used Smart-seq2, STAR alignment to GRCh37/hg19, RSEM quantification and GENCODE v19 annotation (Sade-Feldman et al., 2018). Its deposited processing description specifies exclusion of cells lacking CD45 expression, expressing fewer than 1,000 genes, or having mean housekeeping-gene log2(TPM+1) expression below 2.5. The present analysis began with the deposited quality-controlled matrix of 55,737 genes and 16,291 cells.

The first matrix line contained cell identifiers and the second contained sample labels. Gene-expression rows were parsed after these headers, empty terminal delimiters were trimmed, and each gene row contained 16,291 finite nonnegative values. Cell identifiers were matched to the original metadata. The deposited log2(TPM+1) values were used directly for scoring and marker annotation. All 16,291 cells were retained. Source sample labels carrying T_enriched or myeloid_enriched suffixes were preserved for the enrichment sensitivity analysis.

Broad cell identities were assigned by the largest mean expression of the available genes in these fixed marker panels: T_cell: CD3D, CD3E, CD3G, TRBC1, TRBC2; CD8_T: CD8A, CD8B, CCL5, CD3D; NK: NKG7, GNLY, KLRD1, KLRB1; B_cell: MS4A1, CD79A, CD37, CD74; Myeloid: LYZ, S100A8, S100A9, FCER1G, LST1; Dendritic: FCER1A, CD1C, CLEC10A; Mast: TPSAB1, TPSB2, CPA3. TRBC1 was absent from the deposited gene symbols and therefore omitted from the T-cell denominator; all available/missing memberships are recorded in Supplementary Table S1. The top-two score margin was retained to flag ambiguity. The labels describe broad marker-defined cell populations. The top-scoring label was assigned exclusively to each cell, so T_cell denotes cells assigned to the general T-cell panel and is separate from the CD8_T category in composition summaries. Figure 1F and Supplementary Figure S1 show actual marker-gene expression.

**FIGURE 1:**
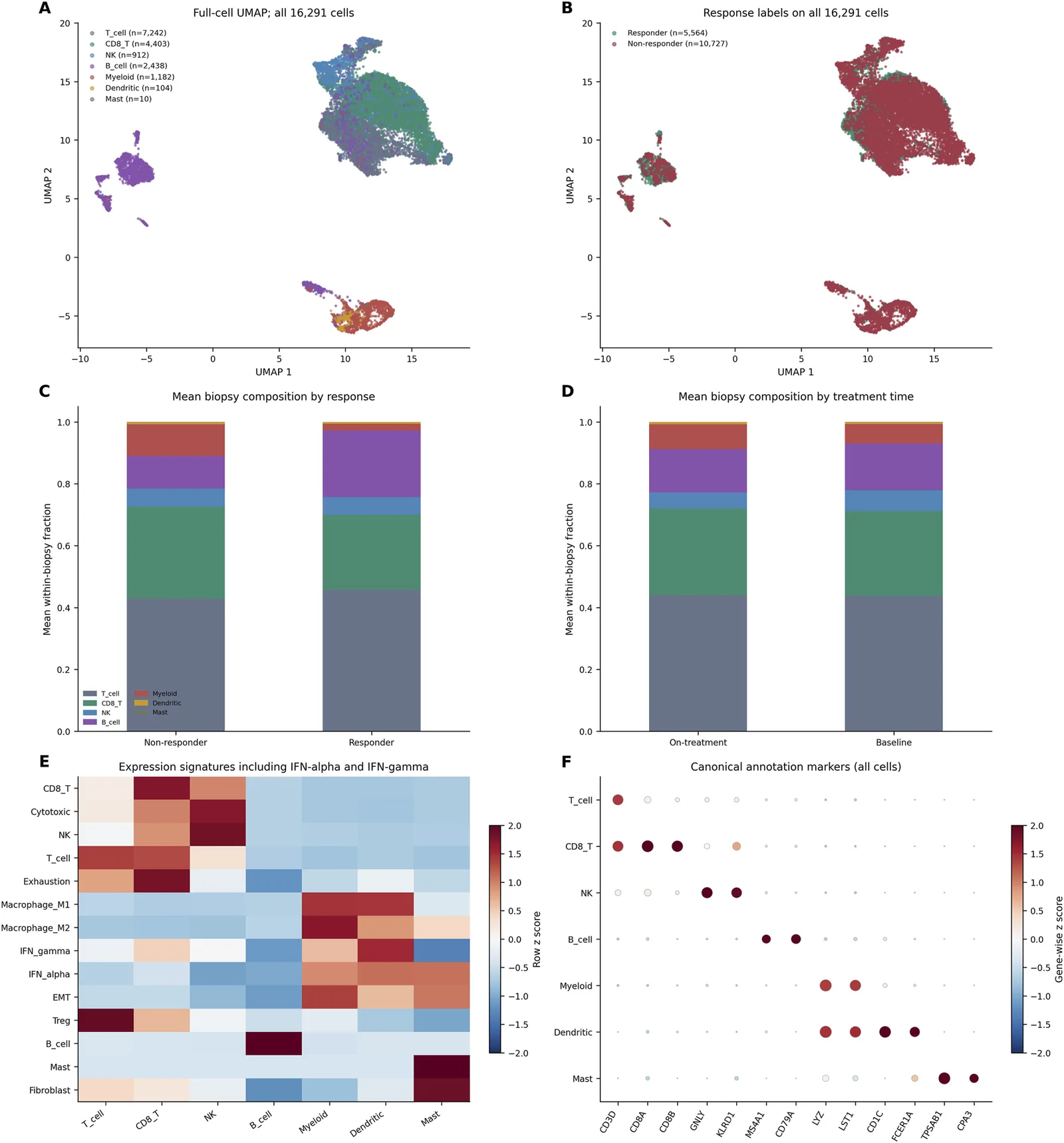
GSE120575 immune-cell landscape. **(A, B)** The same UMAP fitted to and displayed for all 16,291 cells using 2,000 variable genes and 30 PCs, colored by marker-defined cell type or deposited response. **(C, D)** Means of within-biopsy cell fractions by response and treatment time; every biopsy contributes equally and zero-count categories are retained. **(E)** All 14 custom gene-expression signatures, including IFN-α and IFN-γ, calculated from all cells. Colors are row z scores across cell types, showing relative expression within each signature. **(F)** Canonical marker dot plot from all cells; dot area reflects the proportion with expression above zero and color the gene-wise standardized mean. Additional individual marker features are in Supplementary Figure S1. Cell labels denote the marker-defined categories specified in Methods.

For dimensional reduction, genes detected in at least 10 cells were considered; mitochondrial and ribosomal genes were excluded from feature selection only. The top 2,000 mean-bin-standardized variable genes (20 mean bins) were scaled, clipped at ±10, and reduced to 30 PCs. UMAP used all 16,291 cells (20 neighbors, min_dist = 0.3, Euclidean metric, seed = 2026), and every one of these cells was retained in the embedding and all feature plots. All scores, marker summaries and fractions used the full matrix. This simplified rule-based annotation was not based on reference mapping or cell clustering; T_cell and CD8_T were mutually exclusive reporting categories despite biological overlap.

Per-biopsy means of log2(TPM+1) expression were calculated before testing. Each of the 48 biopsies contributed one observation, with repeated biopsies linked to 32 patients. Cell fractions used all cells per biopsy and included zero-count cell types. Regression coefficients estimate responder minus nonresponder differences, adjusted for time point and treatment regimen, with patient-clustered robust covariance, finite-sample correction and t-based inference. BH q values were calculated across the 60 custom signature/checkpoint features, separately across 7 cell fractions, and separately across the two Hallmark scores. Unadjusted Mann–Whitney results are supplied as secondary comparisons. Additional sensitivity analyses comprise baseline-only, on-treatment-only, anti-PD-1-only, removal of explicitly T- or myeloid-enriched cells, and within-cell-type IFN comparisons requiring at least 10 cells per biopsy. Two-sided P values and BH-adjusted q values are reported with effect estimates and 95% confidence intervals.

### 2.5 Cell-line RT-qPCR and statistical analysis

A375 (A-375; ATCC CRL-1619) and SK-MEL-28 (ATCC HTB-72) human melanoma cells were treated with recombinant human IFN-γ at 10 or 100 ng/mL (PeproTech) for 24 or 48 h. Total RNA was extracted and RT-qPCR was performed using the 2^−ΔΔCt^ method with GAPDH as the internal reference. Data are presented as mean ± SD from four independent biological replicates, each measured in technical triplicates. The six Mock-versus-IFN-γ comparisons shown in Figure 2 were assessed using two-sided unpaired Student's t-tests with Holm correction.

**FIGURE 2:**
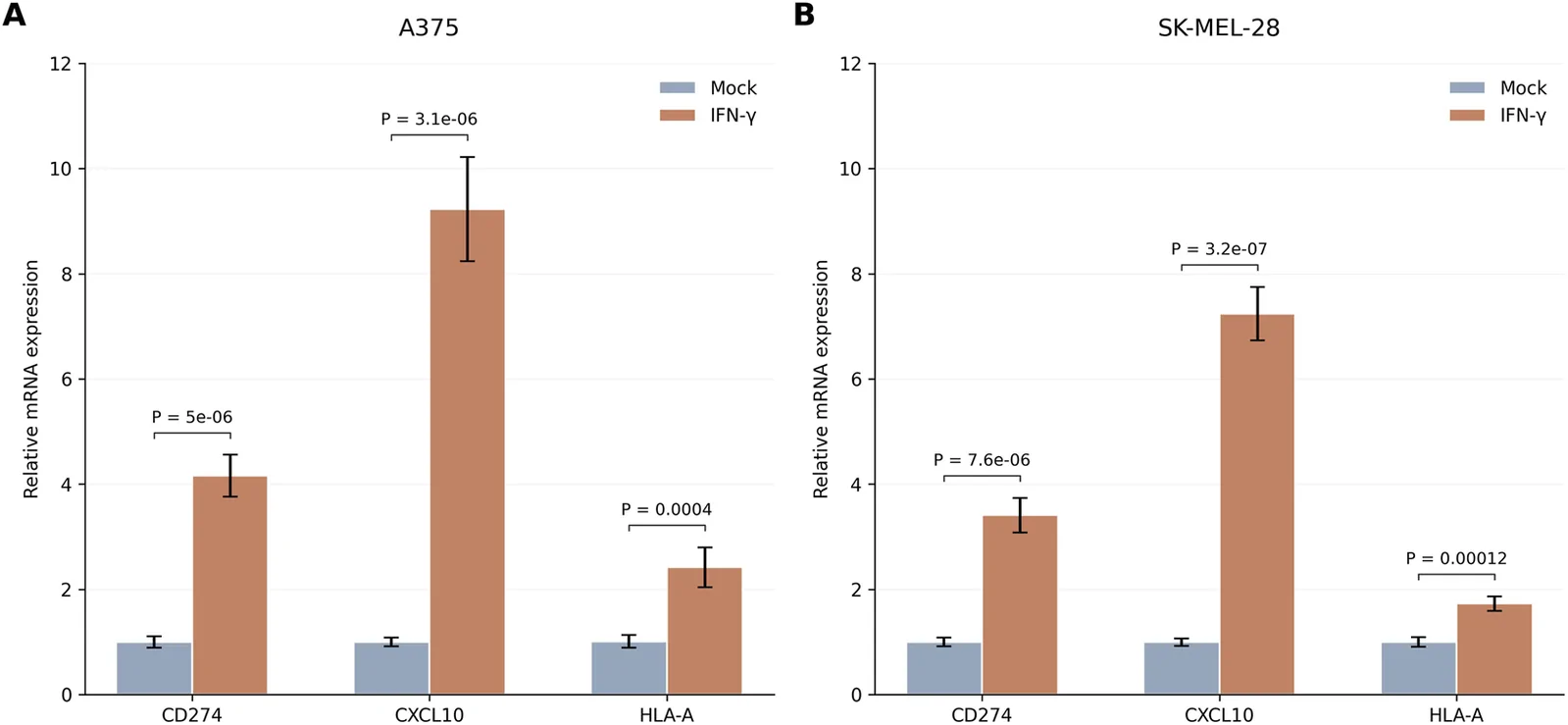
IFN-γ-associated expression of CD274, CXCL10 and HLA-A in **(A)** A375 and **(B)** SK-MEL-28 cells. The experimental protocol used recombinant human IFN-γ at 10 or 100 ng/mL (PeproTech) for 24 or 48 h. RT-qPCR used GAPDH as the internal reference and the 2^(−ΔΔCt)^ method. Bars show the reported relative-expression means and error bars show SD, with four independent biological replicates per group and technical triplicates within each biological replicate. Each bracket compares Mock with IFN-γ for the indicated gene using a two-sided unpaired Student's t-test (df = 6). The displayed P values are approximate values calculated from rounded means and SDs; exact input summaries, mean differences, 95% confidence intervals and Holm-adjusted P values are provided in Supplementary Table S5.

Relative gene expression was calculated for each target using the corresponding mock group as calibrator. For each sample, ΔCt was calculated as Ct(target gene) − Ct(GAPDH), ΔΔCt as ΔCt(sample) − mean ΔCt(mock), and relative fold expression as 2^(−ΔΔCt)^ (Livak and Schmittgen, 2001). The six Mock-versus-IFN-γ comparisons displayed in Figure 2 were evaluated with two-sided unpaired Student's t-tests on the relative-expression scale, using the reported means, SDs and four biological replicates per group (df = 6). Approximate P values were calculated from the rounded summary values; Holm-adjusted P values across the six comparisons are also reported in Supplementary Table S5.

## 3 Results

### 3.1 ICG-based consensus clustering defines three tumor-immune phenotypes in TCGA-SKCM

The heatmap contained 47 checkpoint genes across 472 tumors; median ICG scores were ICG-high = 0.729, ICG-mid = -0.009, ICG-low = -0.674 (Figure 3A). Consensus CDF AUCs were k = 2: 0.501, k = 3: 0.656, k = 4: 0.723, k = 5: 0.780, k = 6: 0.813 (Figure 3B). Successive CDF-area gains were k = 3: 0.155, k = 4: 0.067, k = 5: 0.057, k = 6: 0.033; k = 3 was retained (Figure 3C). Mean consensus was 0.958 within phenotypes and 0.020 between phenotypes (Figure 3D). PC1 and PC2 explained 49.1% and 5.5% of ICG-profile variance (Figure 3E). ICG-high: 136 (28.8%); ICG-mid: 203 (43.0%); ICG-low: 133 (28.2%) (Figure 3F). The gain was greatest at k = 3 (0.155) and smaller at k = 4–6 (0.067, 0.057 and 0.033); the three-cluster solution also showed high within-cluster consensus and interpretable phenotype groups.

**FIGURE 3:**
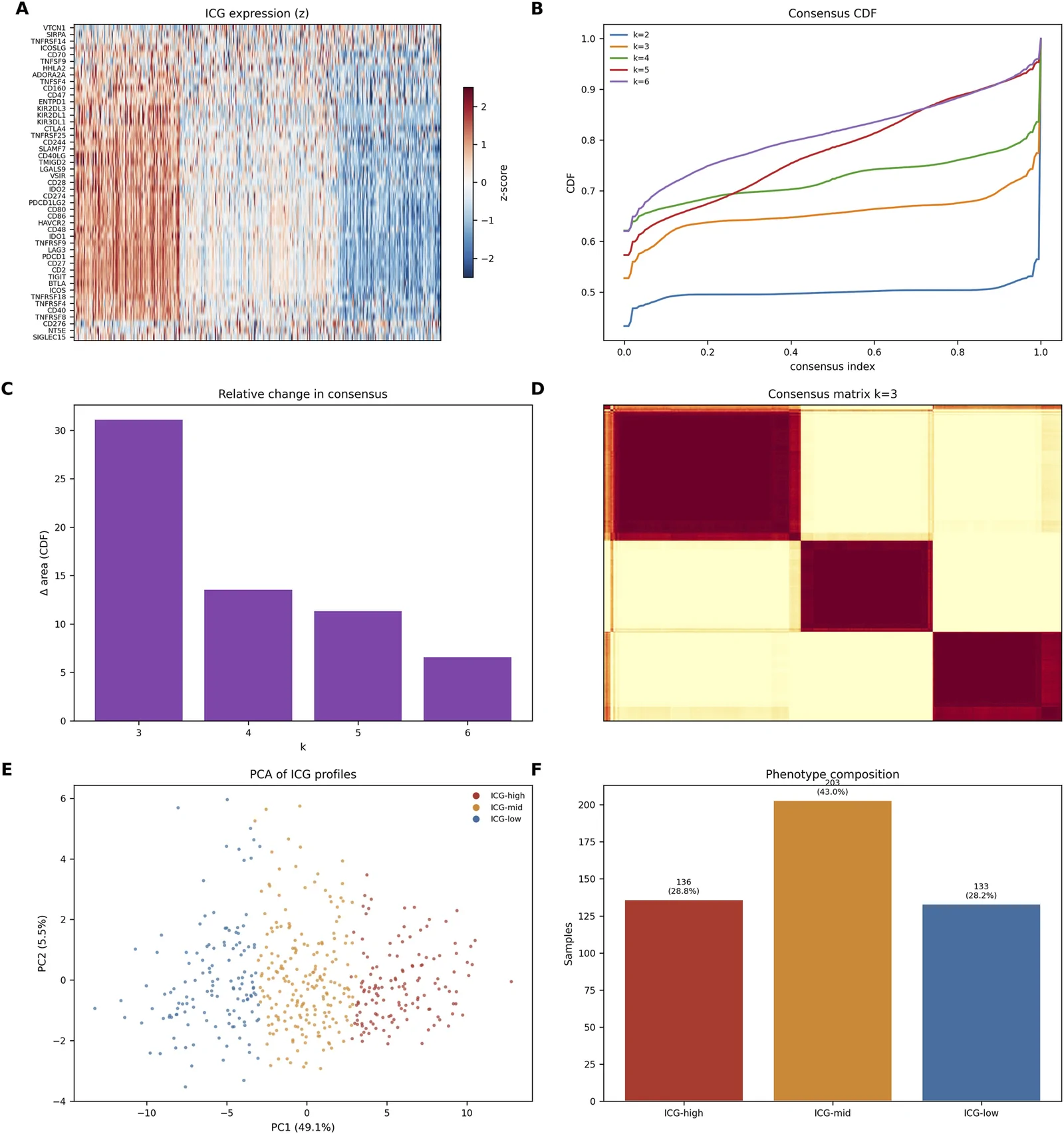
ICG-based phenotypes in TCGA-SKCM. **(A)** Heatmap of 47 ICG z-scores across 472 tumors ordered by phenotype. **(B)** Consensus clustering CDF curves for k = 2–6. **(C)** Relative change in CDF area across k. **(D)** Consensus matrix at k = 3. **(E)** PCA of ICG profiles colored by phenotype. **(F)** Sample counts per phenotype.

### 3.2 Immune microenvironment contexture across ICG phenotypes

Fourteen ssGSEA signatures were summarized; mean CD8 T scores were ICG-high = 0.211, ICG-mid = -0.011, ICG-low = -0.134 (Figure 4A). Six immune-cell scores were summarized; mean CD8 T-cell marker scores were ICG-high = 0.216, ICG-mid = -0.007, ICG-low = -0.150 (Figure 4B). Key signatures separated the phenotypes; the strongest result was T_cell (Kruskal-Wallis P = 3.95e-70) (Figure 4C). Median CD8 T-cell scores were ICG-high = 0.208, ICG-mid = -0.023, ICG-low = -0.160 (Figure 4D). Median ICG scores declined from high to low: ICG-high = 0.729, ICG-mid = -0.009, ICG-low = -0.674 (Figure 4E). ICG score correlated with CD8 T ssGSEA (Spearman rho = 0.828, P = 5.06e-120) (Figure 4F).

**FIGURE 4:**
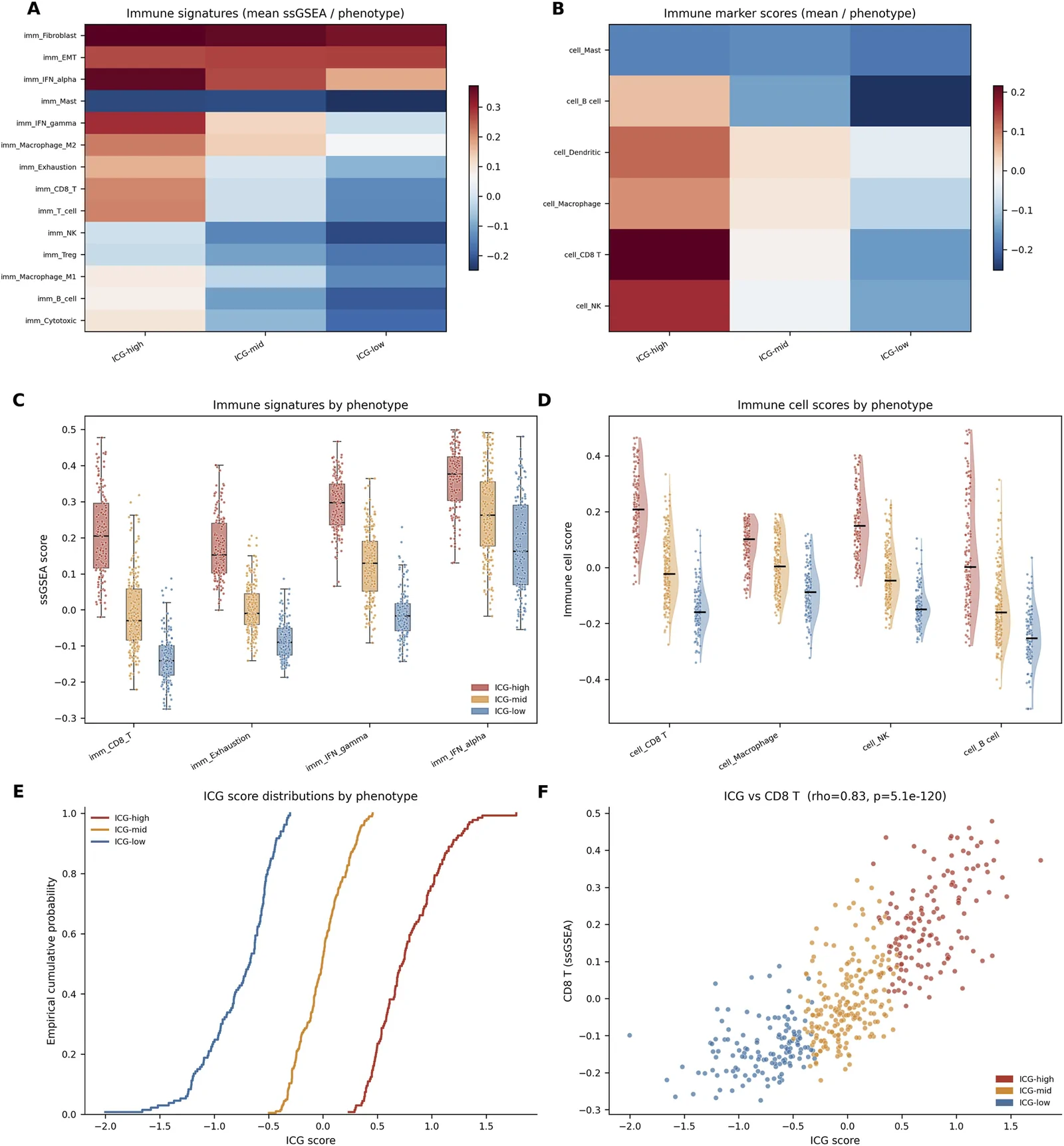
Immune contexture. **(A)** Mean ssGSEA immune signatures per phenotype. **(B)** Mean immune-cell marker scores per phenotype. **(C)** Key immune signature box-and-point distributions. **(D)** Immune-cell marker-score raincloud distributions. **(E)** Empirical cumulative distributions of ICG scores by phenotype. **(F)** ICG score versus CD8 T ssGSEA with Spearman rho.

### 3.3 ICG phenotypes stratify overall survival

After one-tumor-per-patient selection, overall survival differed across ICG phenotypes (454 patients; median OS 4930, 2192, 1487 days for high, mid and low, respectively; log-rank P = 1.50e-05; Figure 5A). Sex and stage survival comparisons used the same patient-level table (Figures 5B,C). The ICG score remained associated with AJCC stage among tumor profiles (Kruskal–Wallis P = 4.43e-05; Figure 5D), motivating covariate adjustment. In 404 complete cases with 190 deaths, adjusted ICG-high versus low was HR 0.34, 95% CI 0.22–0.51; P = 2.68e-07, and ICG-mid versus low was HR 0.57, 95% CI 0.41–0.80; P = 9.18e-04 (Figure 5E). The stage-stratified sensitivity estimate for ICG-high versus low was HR 0.35, 95% CI 0.23–0.53; P = 6.40e-07 (Figure 5F). The ICG-high phenotype was therefore associated with longer overall survival after adjustment for AJCC stage, age and sex. Full coefficients, exclusions and proportional-hazards diagnostics are supplied in Supplementary Table S2.

**FIGURE 5:**
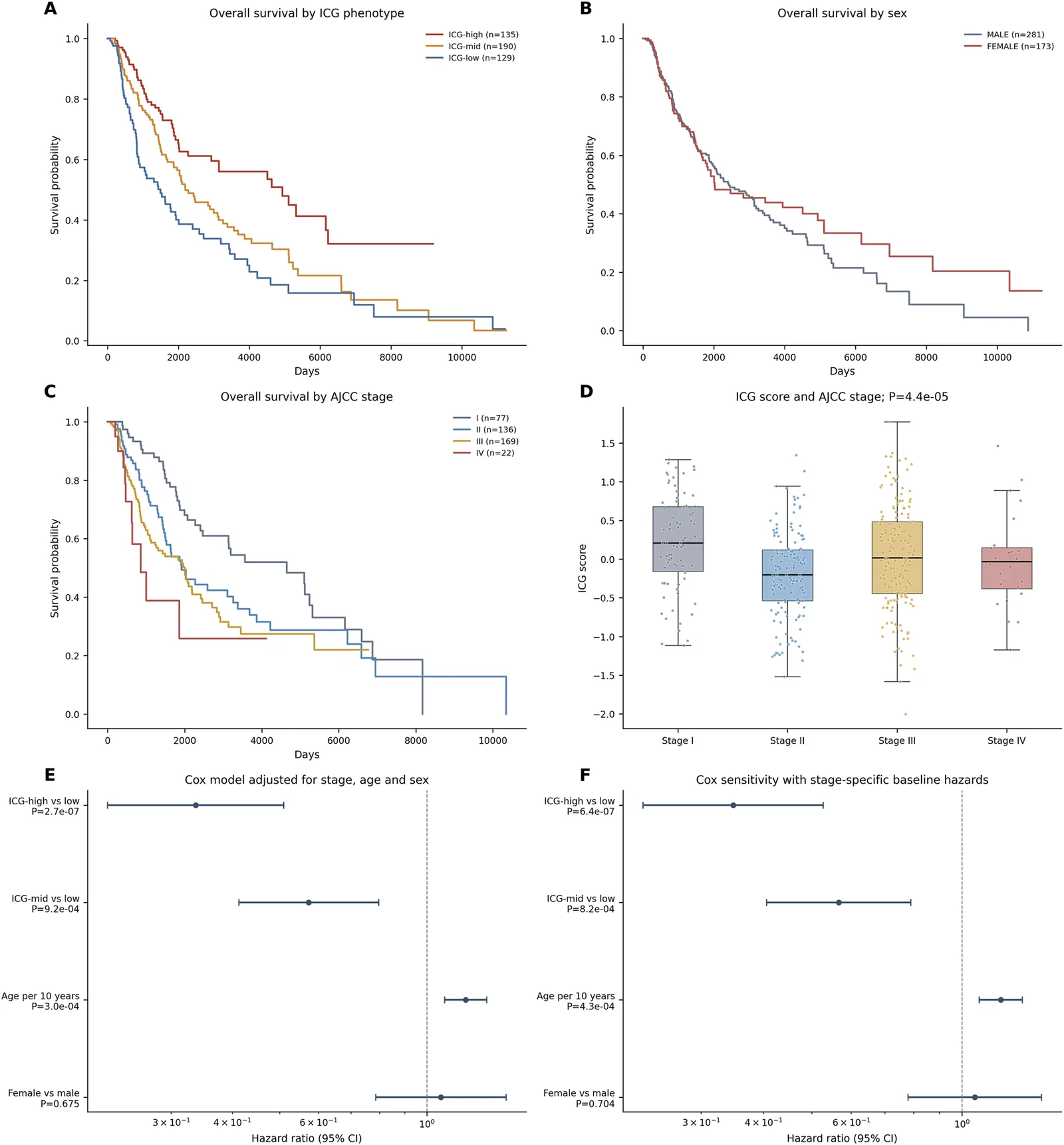
Patient-level overall survival and stage-adjusted associations. **(A)** Kaplan–Meier curves by ICG phenotype, with one tumor per patient. **(B)** Survival by sex. **(C)** Survival by AJCC stage. **(D)** ICG score by stage across tumor profiles. **(E)** Cox estimates adjusted for AJCC stage, age and sex. **(F)** Sensitivity model stratified by AJCC stage. Forest points show hazard ratios and horizontal lines 95% confidence intervals; reference phenotype is ICG-low. Age is per 10 years and the sex reference is male. Sample and event counts, all model terms and proportional-hazards tests are in Supplementary Table S2.

### 3.4 Somatic landscape of ICG phenotypes

BRAF mutation rates were ICG-high = 2/4 (50.0%), ICG-mid = 20/36 (55.6%), ICG-low = 11/26 (42.3%) (Figure 6A). NRAS mutation rates were ICG-high = 1/4 (25.0%), ICG-mid = 5/36 (13.9%), ICG-low = 3/26 (11.5%) (Figure 6B). Overall mutation rates were BRAF = 50.0% and NRAS = 13.6% (Figure 6C). Median total mutation counts were ICG-high = 581.500, ICG-mid = 283.000, ICG-low = 235.000 (Kruskal-Wallis P = 0.261) (Figure 6D). Median UV mutation rates were ICG-high = 0.896, ICG-mid = 0.845, ICG-low = 0.836 (Kruskal-Wallis P = 0.131) (Figure 6E). The oncoprint included 66 samples and 17 mutation fields; BRAF and NRAS accounted for 33 and 9 mutated samples (Figure 6F). These mutation comparisons are exploratory because annotations were available for only 66 tumors, including four in the ICG-high group.

**FIGURE 6:**
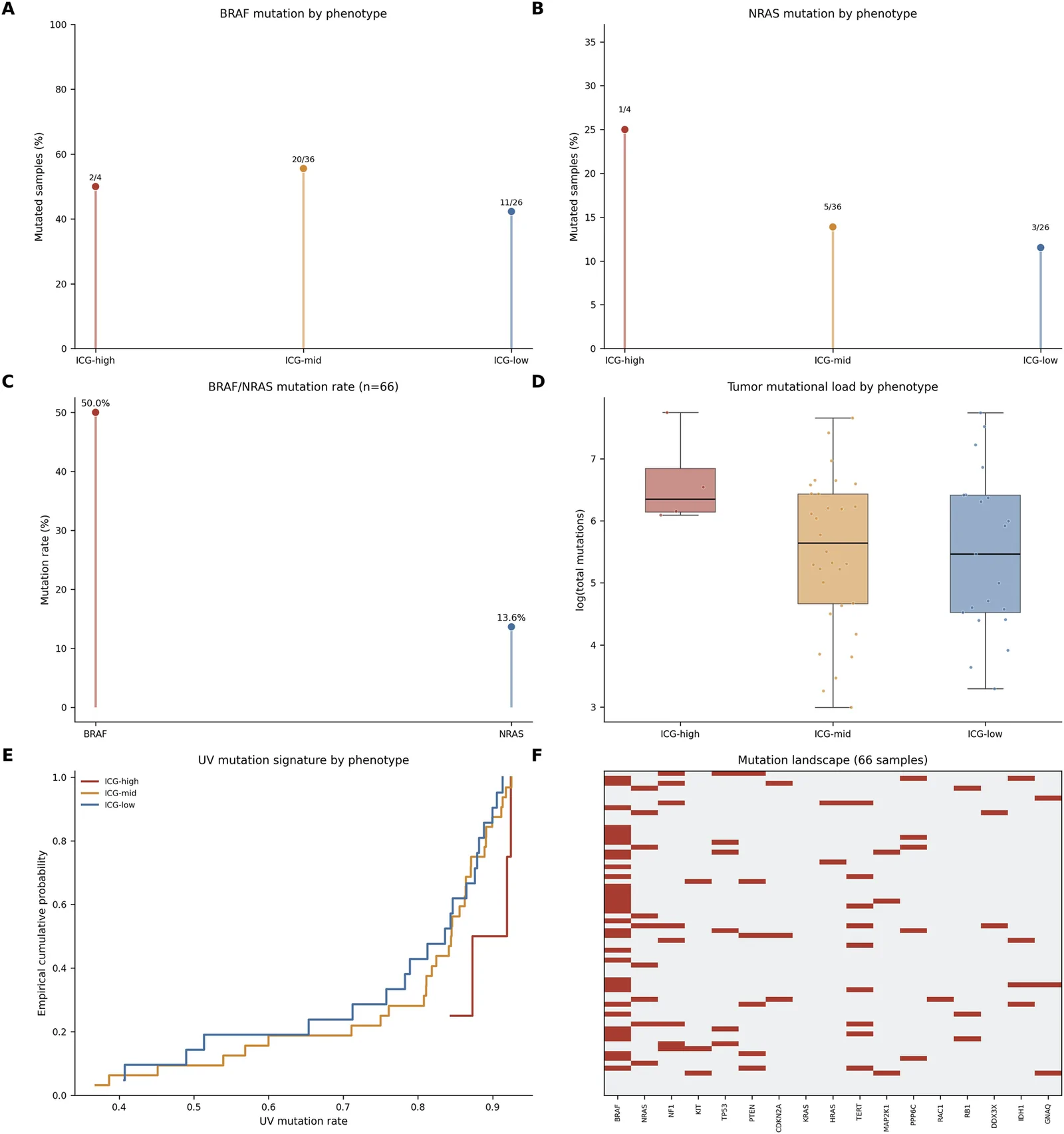
Somatic landscape. **(A)** BRAF mutation rate by phenotype shown as point-and-stem fractions. **(B)** NRAS mutation rate by phenotype shown as point-and-stem fractions. **(C)** Overall BRAF/NRAS mutation rates shown as a point-and-stem comparison. **(D)** Total mutational load box-and-point distributions on the log1p scale. **(E)** UV mutation-rate empirical cumulative distributions by phenotype. **(F)** Mutation landscape of 66 annotated samples. These exploratory analyses included only 66 mutation-annotated tumors, including four ICG-high tumors.

### 3.5 On-treatment immune remodeling in GSE91061

Median ICG score changed from −0.105 before treatment to 0.051 on treatment (median delta = 0.115, paired Wilcoxon P = 0.012) (Figure 7A). Median CD8 T changed from 0.142 before treatment to 0.209 on treatment (median delta = 0.041, paired Wilcoxon P = 1.93e-04) (Figure 7B). Median IFN-γ changed from 0.304 before treatment to 0.358 on treatment (median delta = 0.048, paired Wilcoxon P = 0.001) (Figure 7C). Median exhaustion changed from 0.047 before treatment to 0.115 on treatment (median delta = 0.021, paired Wilcoxon P = 0.001) (Figure 7D). ICG-score delta had median 0.115 and IQR -0.061 to 0.391 (Figure 7E). The strongest paired change was Cytotoxic (median delta = 0.031, P = 1.23e-04); 10/10 top features increased (Figure 7F).

**FIGURE 7:**
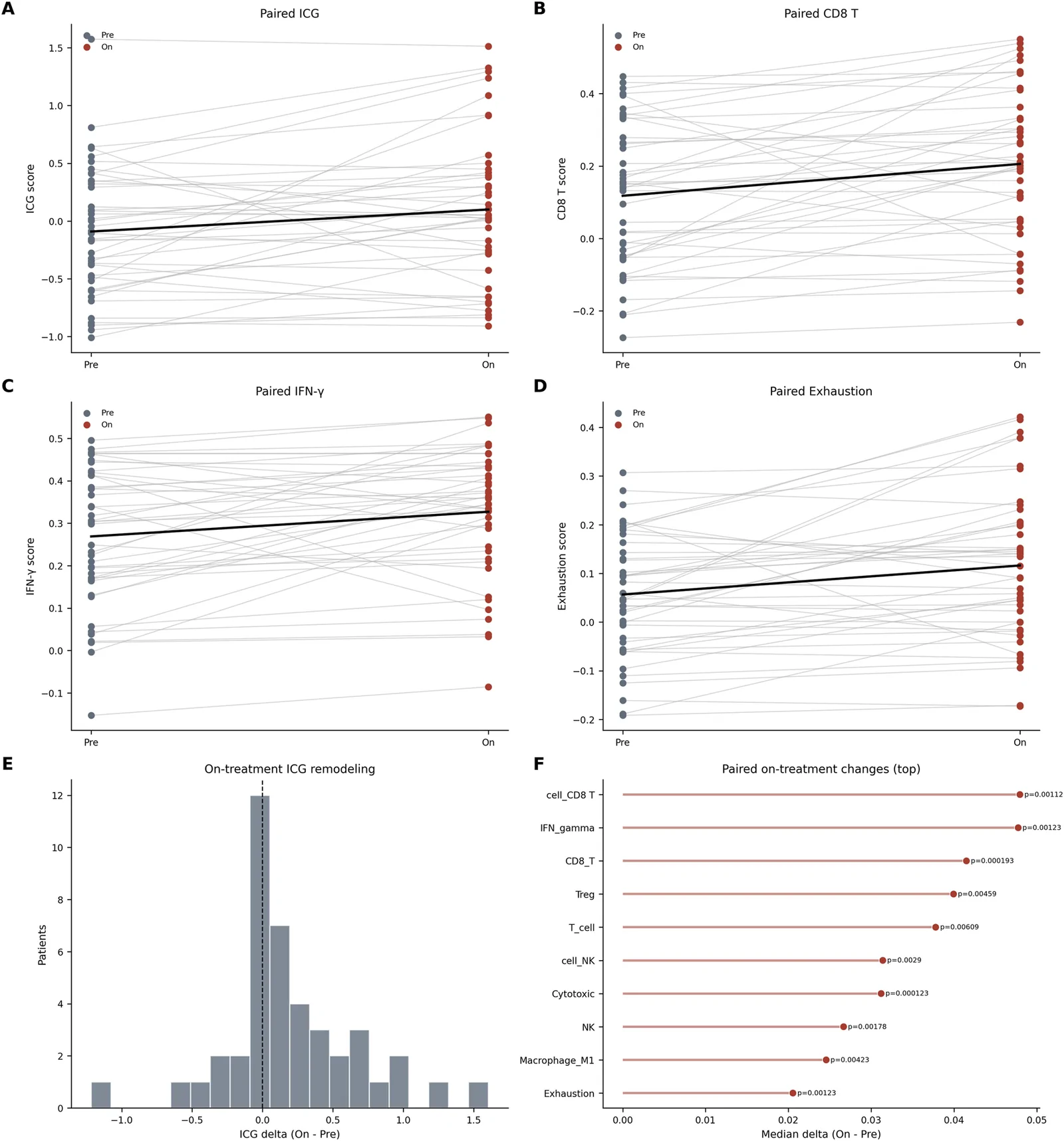
On-treatment remodeling in GSE91061. **(A–D)** Paired pre/on-treatment ICG score, CD8 T, IFN-γ, and exhaustion scores; gray lines are individual patients, black line the mean. **(E)** Distribution of on-treatment ICG delta. **(F)** Paired on-treatment changes shown as point-and-stem median deltas with paired Wilcoxon P.

### 3.6 Immune signature architecture in the immunotherapy cohort

Fourteen ssGSEA signatures were displayed across 109 samples (51 pre-treatment; 58 on-treatment) (Figure 8A). Six immune-cell marker scores were displayed across the same 109 samples (Figure 8B). Median ICG scores by visit were Pre = -0.155, On = 0.052 (Figure 8C). On-minus-pre median differences were imm_CD8_T = 0.081, imm_Exhaustion = 0.090, imm_IFN_gamma = 0.096 (Figure 8D). PC1 and PC2 explained 71.6% and 10.6% of immune-signature variance (Figure 8E). The strongest off-diagonal ICG correlation was CD86-HAVCR2 (rho = 0.973) (Figure 8F). The imm_IFN_gamma value 0.096 denotes the difference between the two visit-group medians. The paired IFN-γ change is 0.048 in 43 patients (Figure 7C). Both comparisons concern pretreatment versus on-treatment expression.

**FIGURE 8:**
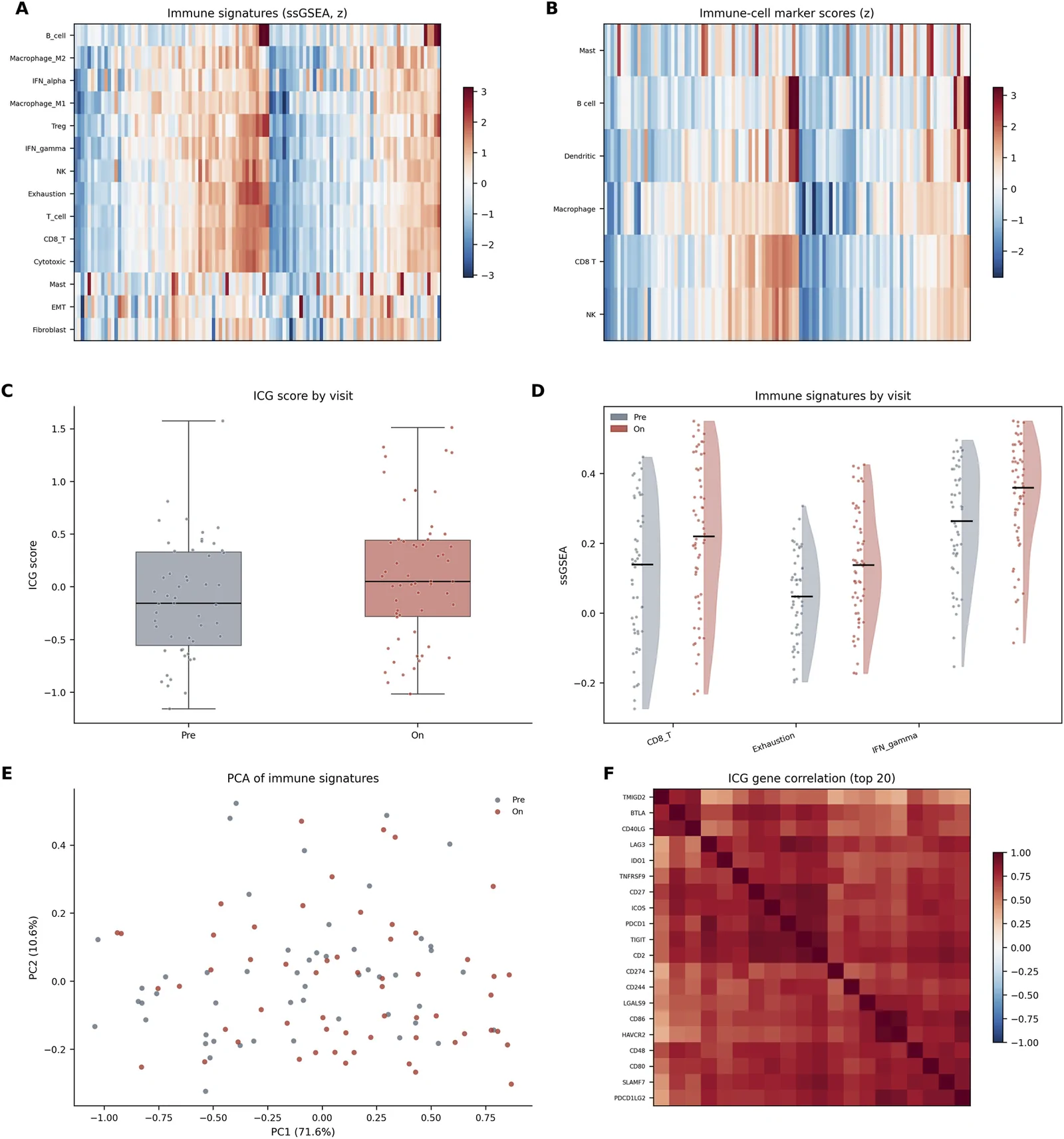
Immunotherapy cohort signatures. **(A)** Immune signature heatmap (z). **(B)** Immune-cell marker-score heatmap (z). **(C)** ICG score box-and-point distributions by visit. **(D)** Immune-signature raincloud distributions by visit. **(E)** PCA of immune signatures by visit. **(F)** ICG gene correlation heatmap (top 20 genes).

#### 3.6.1 Single-cell immune landscape (GSE120575)

All 16,291 cells were retained after strict matrix and metadata alignment, and all cells were used in the embedding, feature plots, scoring and composition summaries (Figures 1A,B). Marker-based annotation yielded T_cell = 7242, CD8_T = 4403, B_cell = 2438, Myeloid = 1182, NK = 912, Dendritic = 104, Mast = 10. Response and time-point composition plots summarize mean within-biopsy fractions calculated from all cells (Figures 1C,D), giving each biopsy equal descriptive weight. Figure 1E displays all 14 custom signatures, including IFN-α and IFN-γ; its row-standardized colors compare cell types within each signature using a separate standardized scale for each row. The Macrophage_M1 panel includes NOS2, TNF, IL1B, CXCL9, CXCL10 and CD80; its signal in dendritic cells represents expression of this inflammatory marker panel. Figure 1F and Supplementary Figure S1 display marker-gene expression supporting the marker-based annotations. The matrix includes 873 explicitly T-enriched and 118 myeloid-enriched cells; their contribution was examined by an additional analysis excluding these enrichment-labelled cells.

### 3.7 Single-cell features associated with immunotherapy response

Among 48 biopsies from 32 patients, 20 responder biopsies represented 14 patients and 28 nonresponder biopsies represented 18 patients. On the deposited log-expression scale, biopsy IFN-α scores were 1.343 versus 2.084 (responders versus nonresponders; adjusted difference −1.147, 95% CI -1.683 to −0.612; P = 1.29e-04, q = 0.004); IFN-γ scores were 1.104 versus 1.657 (responders versus nonresponders; adjusted difference −0.556, 95% CI -0.921 to −0.191; P = 0.004, q = 0.025). Both observed directions remained higher in nonresponders after adjustment for time point and therapy with patient-clustered inference (Figures 9A,B). Baseline and on-treatment distributions are shown separately (Figure 9C), while checkpoint expression and zero-inclusive biopsy fractions appear in Figures 9D,E. Fixed IFN sensitivity analyses, including within-cell-type estimates, are reported in Figure 9F and Supplementary Table S4. The baseline-only IFN-γ estimate was −0.533 (95% CI -1.078 to 0.013; P = 0.055; 19 biopsies), and the on-treatment estimate was −0.592 (95% CI -1.044 to −0.140; P = 0.012; 29 biopsies). Official Hallmark IFN-α scores were 1.954 versus 2.526 (responders versus nonresponders; adjusted difference −0.712, 95% CI -1.005 to −0.420; P = 2.36e-05, q = 2.52e-05); Hallmark IFN-γ scores were 1.758 versus 2.386 (responders versus nonresponders; adjusted difference −0.631, 95% CI -0.891 to −0.371; P = 2.52e-05, q = 2.52e-05). These estimates describe biopsy-level IFN expression in the immune-cell compartment by response group.

**FIGURE 9:**
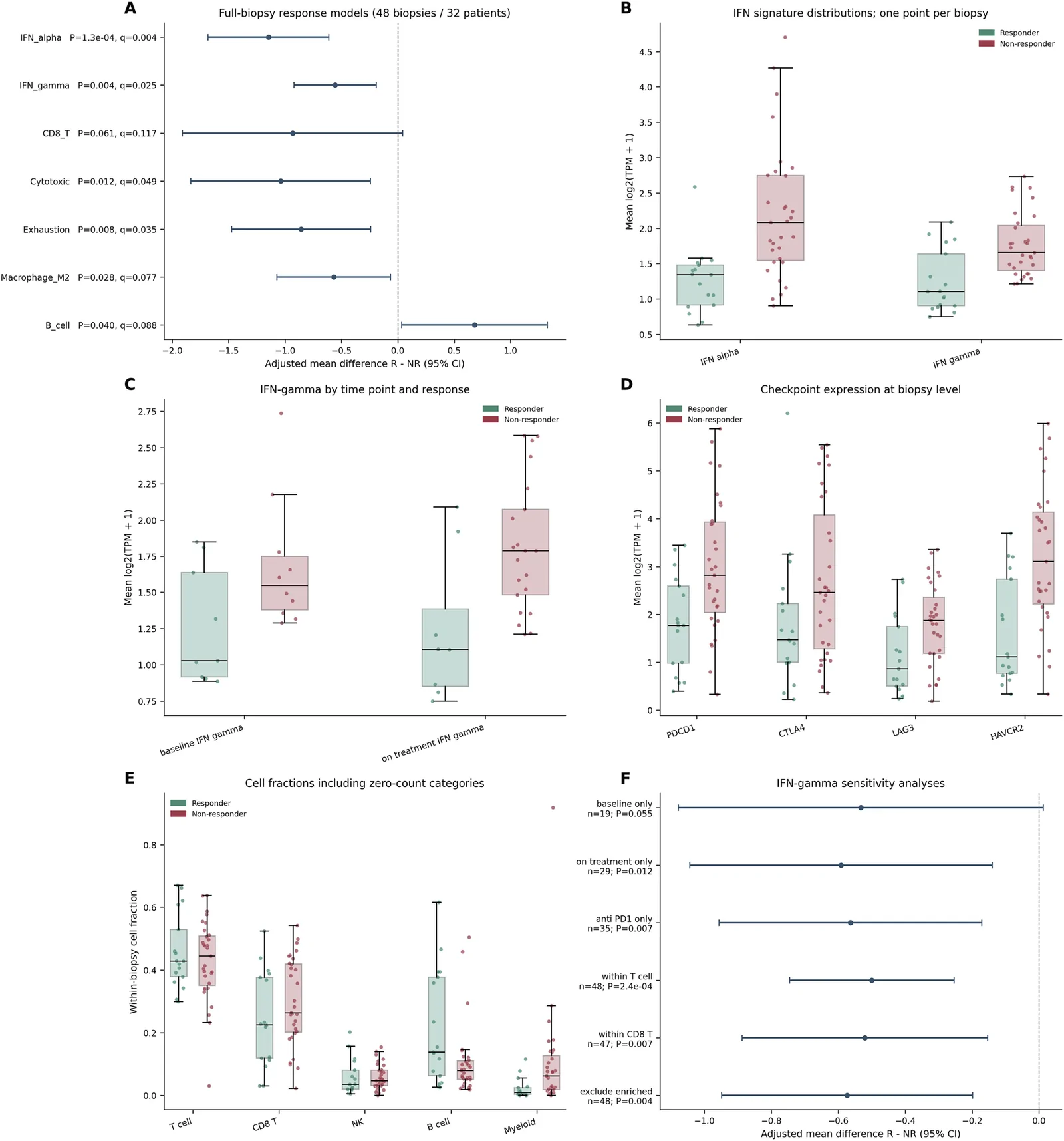
Patient-aware single-cell response comparisons using all eligible cells. **(A)** Selected fixed signature coefficients for responder minus nonresponder from biopsy-level models adjusted for time point and therapy; bars are 95% confidence intervals, P values use patient-clustered covariance and q values use BH correction across all 60 tested features. **(B)** Both IFN signature distributions. **(C)** IFN-γ shown separately at baseline and on-treatment. **(D)** Checkpoint expression. **(E)** Cell fractions normalized within each biopsy, including zeros. In B–E, each point is a biopsy; boxes show medians and interquartile ranges, whiskers 1.5 times the interquartile range. **(F)** Fixed IFN-γ sensitivities; n denotes biopsies, and each model accounts for repeated patient records where present. Gene-expression units are deposited log2(TPM+1).

#### 3.7.1 qPCR results

IFN-γ treatment was associated with increased relative expression of CD274, CXCL10, and HLA-A in both melanoma cell lines (Figure 2). In A375 cells, relative CD274 expression increased from 1.00 ± 0.11 in the mock condition to 4.16 ± 0.40 following IFN-γ treatment. CXCL10 showed the largest increase, from 1.00 ± 0.08 to 9.23 ± 0.99, whereas HLA-A expression increased from 1.01 ± 0.12 to 2.42 ± 0.38. A similar expression pattern was observed in SK-MEL-28 cells. Relative CD274 expression increased from 1.00 ± 0.08 in the mock group to 3.41 ± 0.33 following IFN-γ treatment, while CXCL10 expression increased from 1.00 ± 0.07 to 7.24 ± 0.51. HLA-A showed a more modest increase, from 1.00 ± 0.09 in the mock condition to 1.73 ± 0.14 following IFN-γ treatment. Overall, the qPCR results showed higher relative expression of all three genes following IFN-γ treatment in both A375 and SK-MEL-28 cells, with the largest fold changes observed for CXCL10. Data are presented as mean ± SD from four independent biological replicates per group, each measured in technical triplicate. The two-sided unpaired Student's t-tests gave A375: CD274 P = 5.05e-06, CXCL10 P = 3.08e-06, HLA-A P = 0.000399; SK-MEL-28: CD274 P = 7.64e-06, CXCL10 P = 3.24e-07, HLA-A P = 0.000122. All six Holm-adjusted P values were below 0.001 (Supplementary Table S5).

## 4 Discussion

The ICG phenotype association with survival persisted after adjustment for measured AJCC stage, age and sex in 404 TCGA complete cases (ICG-high versus low: HR 0.34, 95% CI 0.22–0.51; P = 2.68e-07). A stage-stratified model gave a similar interpretation (HR 0.35, 95% CI 0.23–0.53; P = 6.40e-07). The concordant estimates support an association between ICG phenotype and overall survival after adjustment for these clinical covariates.

The positive association between checkpoint-gene expression and T-cell signatures need not imply a simple relation between increased checkpoint expression and stronger immune suppression. Immune activation can coexist with compensatory inhibitory signaling, as discussed in established frameworks of adaptive immune resistance (Ribas and Wolchok, 2018; Sharma et al., 2017). In the present analysis, the mean CD8 T-cell ssGSEA score decreased from 0.211 in ICG-high to −0.134 in ICG-low, and the correlation between ICG and CD8 T-cell scores was substantial. Bulk transcriptomic signatures describe the coordinated expression of checkpoint and T-cell programs (Galon and Bruni, 2019).

ICG-low tumors exhibited lower CD8 T-cell signature scores in the TCGA analysis, suggesting a distinct transcriptional immune context. M2 macrophage, exhaustion and epithelial–mesenchymal transition scores provide additional descriptions of this context (Sharma et al., 2017; Galon and Bruni, 2019). These expression patterns help frame the biological heterogeneity relevant to combination immunotherapy (Tawbi et al., 2022).

The somatic-mutation analysis included the 66 tumors with available mutation annotations. Median total mutation counts were 581.5, 283.0 and 235.0 in ICG-high, ICG-mid and ICG-low tumors, respectively (Kruskal–Wallis P = 0.261). Four mutation-annotated tumors belonged to ICG-high. The mutation results describe available gene alterations and total mutation counts in this subset (Anagnostou et al., 2022; Chan et al., 2019).

The GSE91061 analysis adds a treatment-associated temporal dimension. Among 43 paired patients, the median ICG score increased during treatment (median paired change = 0.115, Wilcoxon P = 0.012), together with increases in CD8 T-cell, IFN-γ and exhaustion signatures. Concurrent increases in effector and exhaustion signals are consistent with immune activation accompanied by regulatory feedback during nivolumab treatment (Riaz et al., 2017; Sharma et al., 2017; Hugo et al., 2016).

The IFN findings concern four complementary comparisons. TCGA relates bulk ICG phenotypes to overall survival in a heterogeneous cohort and therefore estimates prognosis; it does not test whether ICG or IFN predicts benefit from immune checkpoint blockade. GSE91061 measures within-patient changes in bulk tumor transcriptomes from baseline to nivolumab treatment, whereas GSE120575 compares CD45-positive immune-cell expression between response groups at the biopsy level. The qPCR experiment measures acute IFN-γ inducibility in melanoma cell lines. These designs use different patients, compartments, time points and outcomes, so their effect directions are not expected to form one monotonic axis. In GSE120575, the baseline-only IFN-γ estimate was directionally lower in responders but imprecise (P = 0.055), while the on-treatment estimate was more precise and remained lower in responders (P = 0.012). This pattern is compatible with an on-treatment IFN-high or feedback-regulated immune state in nonresponders, but it does not establish that treatment caused the difference. Acute IFN-γ signaling can induce antigen-presentation and checkpoint molecules in melanoma cells (Luo et al., 2018), whereas sustained tumor-cell interferon signaling has been linked experimentally to adaptive resistance (Benci et al., 2016). The qPCR induction of CD274, CXCL10 and HLA-A therefore confirms pathway responsiveness in melanoma cells; it does not imply that IFN-high immune-cell biopsies should have better clinical response. Thus, higher IFN scores in nonresponders may reflect persistent immune stimulation or resistance-associated feedback rather than effective tumor control. The direction persisted after time-point and therapy adjustment and with the official Hallmark sets, but the single-cell analysis remains an association and does not establish causality.

The analysis combines bulk tumor expression, marker-defined immune-cell populations and melanoma-cell stimulation to examine IFN and checkpoint programs at distinct biological levels. Patient-level survival modeling, biopsy-level response comparisons, full-cell visualization, explicit gene membership and prespecified sensitivity analyses provide the basis for these comparisons.

### 4.1 Study limitations

The cohorts were not linked at the patient level, treatment regimens and sampling times differed, and GSE120575 profiled CD45-positive immune cells rather than the tumor compartment. The single-cell response analysis included 48 biopsies from 32 patients; repeated-patient clustering and sensitivity analyses reduce dependence on individual biopsies, while the imprecise baseline-only estimate shows that pretreatment separation is uncertain. Cell composition may contribute to biopsy-level IFN differences despite within-cell-type analyses. TCGA survival is a prognostic association and cannot establish predictive value for ICI benefit, and the qPCR experiment validates acute pathway inducibility rather than clinical response. These constraints support interpreting IFN direction as context-specific association rather than a universal response biomarker.

## 5 Conclusions

Melanoma ICG phenotypes were associated with distinct immune-expression profiles and overall survival after adjustment for AJCC stage, age and sex. Paired bulk profiles showed increased IFN-related expression during treatment, while immune-cell IFN scores were higher in nonresponders in the single-cell cohort. Analysis of all 16,291 cells, biopsy-level statistical comparisons and documented marker and gene-set definitions clarify the cellular and clinical contexts of these findings.

## Data Availability

The datasets presented in this study can be found in online repositories. The names of the repository/repositories and accession number(s) can be found in the article/Supplementary Material.

## Supplementary material

SUPPLEMENTARY FIGURE S1 Canonical marker feature plots on the Figure 7 embedding. Each point is one of all 16,291 cells, colored by its deposited log2(TPM+1) expression. Color scales are clipped at each gene’s 99th full-cell percentile for visibility. Genes are CD3D, CD8A, CD8B, NKG7, GNLY, MS4A1, CD79A, LYZ, LST1, CD1C, FCER1A and TPSAB1.

SUPPLEMENTARY TABLES S1 Exact custom and annotation gene memberships, official Hallmark JSON records and coverage. S2: TCGA patient selection, complete cases, Cox coefficients and proportional-hazards diagnostics. S3: single-cell preprocessing audit, full cell annotations and full-cell coverage and per-panel cell-count audit. S4: full biopsy scores, zero-inclusive fractions, adjusted response estimates and IFN sensitivity analyses. S5: qPCR experimental conditions, replicate structure, statistical tests and summary results. Tables are supplied as machine-readable source tables with an index.
